# Supplementary material for: Long-range GABAergic projections contribute to cortical feedback control of sensory processing
Source: Nat Commun. 2022 Nov 12;13:6879. doi: 10.1038/s41467-022-34513-0 (PMC9653434; doi:10.1038/s41467-022-34513-0)
Supplement: Supplementary file 1 — Supplementary Information [file 41467_2022_34513_MOESM1_ESM.pdf]

Long-range GABAergic projections contribute to cortical feedback control of sensory processing.

Camille Mazo[1, 2, *], Antoine Nissant[1], Soham Saha[1], Enzo Peroni[1], Pierre-Marie Lledo[1, #, *] and Gabriel Lepousez[1, #, *]

[1] Institut Pasteur, Université Paris Cité, Centre National de la Recherche Scientifique, Unité Mixte de Recherche 3571, Perception and Memory Unit, F-75015 Paris, France

Supplementary Information (10 figures and 1 table)

Supplementary Fig. 1. Further characterization of the anterograde labeling.

Supplementary Fig. 2. Further anatomical and neurochemical analysis of the OB-projecting GABAergic cells in the AON/APC.

Supplementary Fig. 3. Further characterization of the OB-projecting GABAergic cells.

Supplementary Fig. 4. The primary somatosensory cortex (S1) also sends GABAergic projections back to the somatosensory thalamus nuclei.

Supplementary Fig. 5. Monosynaptic retrogradely-labeled neurons from genetically identified OB neuron populations.

Supplementary Fig 6. Cortico-bulbar GABAergic axon stimulation increases beta oscillations.

Supplementary Fig. 7. Further characterization of cortical GABAergic inhibition in the OB neuron populations.

Supplementary Fig. 8. Additional analysis of cortico-bulbar inhibition of OB output neurons.

Supplementary Fig. 9. Additional analysis of the behavioral effects of GABAergic cortico-bulbar axon silencing.

Supplementary Fig. 10. Protocol for neuronal activity data extraction using awake 2-photon imaging.

Supplementary Table 1. Membrane resistance (Rm) and kinetics of the light-evoked IPSCs in the post-synaptic neurons.

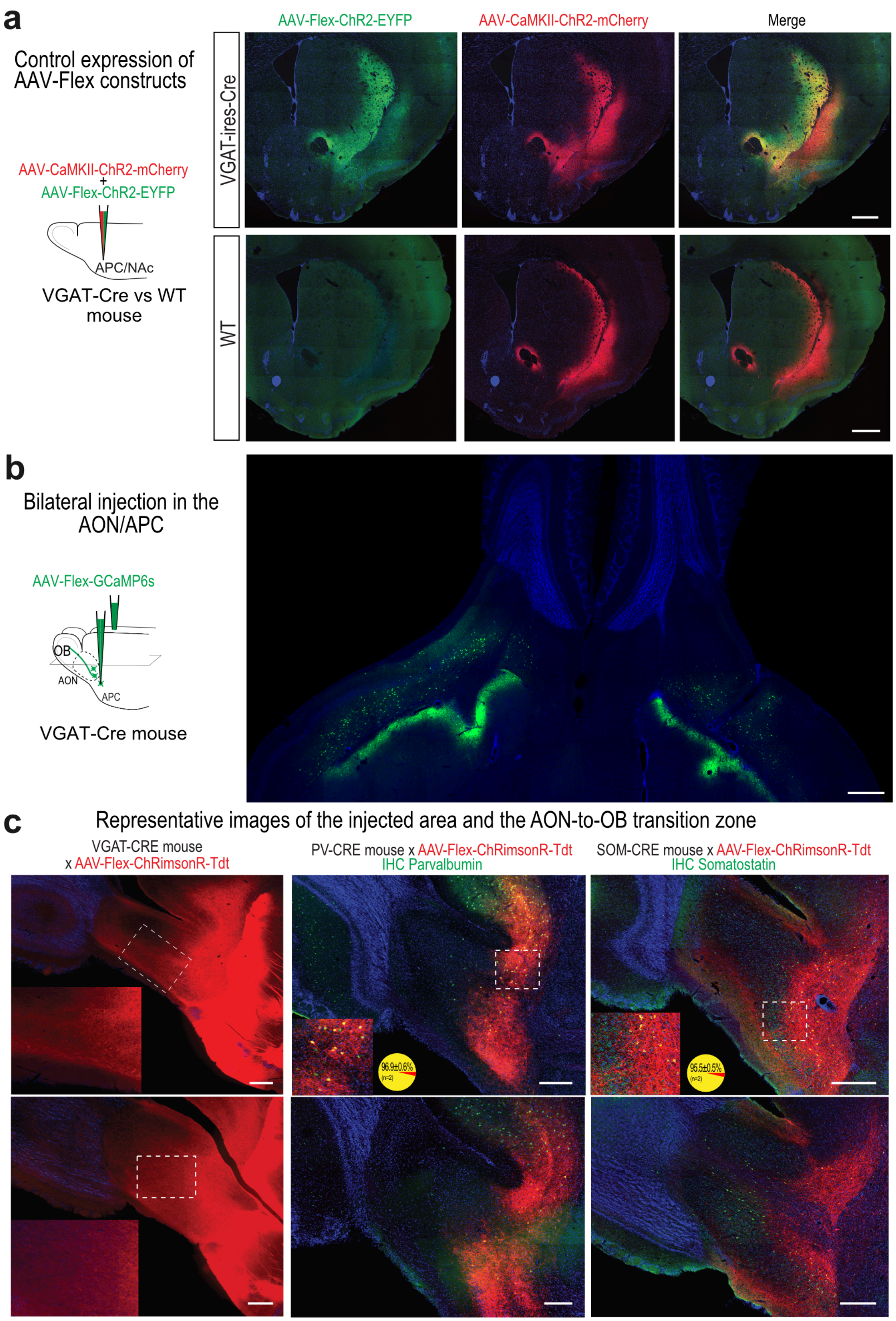

Supplementary Fig. 1. Further characterization of the anterograde labeling. a, Cre-dependent virus specificity control. Injection of a mix of Cre and non-Cre dependent viruses (AAV-Flex-ChR2-eYFP and AAV-

CaMKII-Chr2-mCherry, respectively) were injected in VGAT-Cre (n=2 animals) versus WT mice (n=2 animals). Three weeks post injection, no labeling associated with the Cre-dependent virus was observed in WT mice. Co-injection of the non-Cre dependent virus assured the proper targeting of the injection. Injections were targeted in the APC as well as in the nucleus accumbens (NAc) as the latter contains a high proportion of GABAergic neurons compared to the cortex. This observation was confirmed in n=2 animals. **b,** Representative example of an horizontal slice showing the bilateral injection sites in the AON/APC of VGAT-Cre mice. **c,** Representative images of the injection site in VGAT-Cre (left), PV-IRES-Cre (middle) and SOM-IRES-Cre (right) mice. Top and bottom rows are different parasagittal planes of the same mice. Bottom left are insets of the dashed boxes in the same image. In VGAT-Cre mice, the virus only invaded the posterior part of the peduncle and did not diffuse in the OB. Note the lack of neurons infected in the Rostral Migratory Stream (top). We confirmed the specificity of the mouse lines and the efficiency of our IHC staining protocols against PV and SOM markers in PV-IRES-Cre and SOM-IRES-Cre mice, respectively. Colocalizations percentages are percentages of virally-labeled cells that were co-labeled with IHC for the corresponding marker (n = 2 mice per genotype). Scale bars are 500 µm (**a, b**) and 250 µm (**c**).

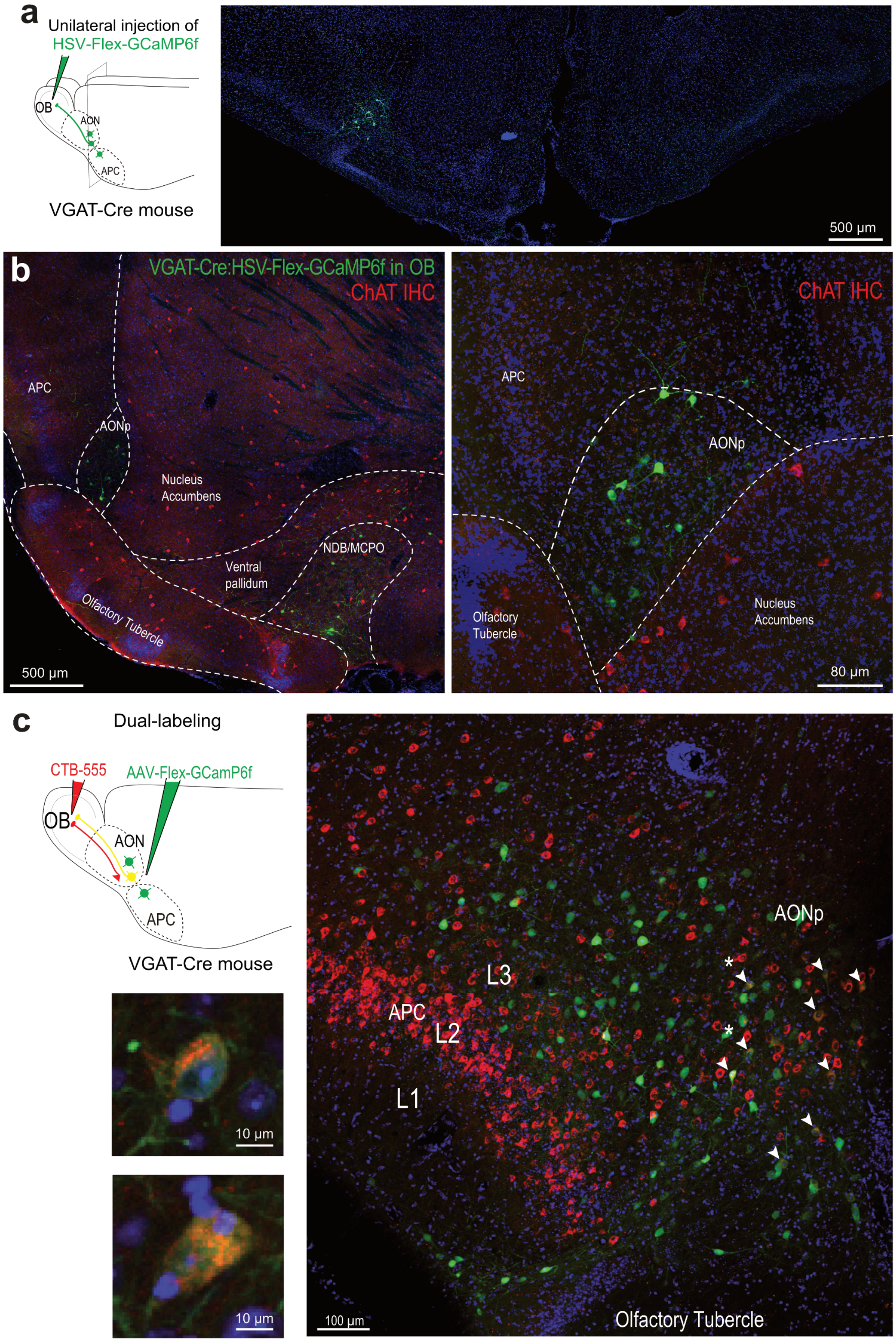
a
Unilateral injection of
HSV-Flex-GCaMP6f
OB
AON
APC
VGAT-Cre mouse
500 µm
b
VGAT-Cre:HSV-Flex-GCaMP6f in OB
ChAT IHC
APC
AONp
Nucleus
Accumbens
NDB/MCPO
Ventral
pallidum
Olfactory Tubercle
500 µm
ChAT IHC
APC
AONp
Olfactory
Tubercle
Nucleus
Accumbens
80 µm
c
Dual-labeling
CTB-555
AAV-Flex-GCamP6f
OB
AON
APC
VGAT-Cre mouse
10 µm
10 µm
AONp
L3
APC
L2
L1
Olfactory Tubercle
100 µm

Supplementary Fig. 2. Further anatomical and neurochemical analysis of the OB-projecting GABAergic cells in the AON/APC. **a,** Representative unilateral OB injection of the retrograde virus HSV-Flex-GCaMP6f labeled OB-projecting GABAergic neurons ipsilaterally, but not contralaterally in the AON and APC (no cell observed in the contralateral hemisphere of n=4/4 animals). **b**, Representative example of Choline acetyltransferase (ChAT) IHC in a AONp-containing brain section showing that OB retrogradely-labeled GABAergic cells are located outside the cholinergic-rich brain area (compare with cells in the NDB/MCPO; this observation was confirmed in n=4 animals). This argues against the hypothesis that this region is a rostral extension of a striatal or pallidal structure. **c**, Representative example of non-selective retrograde labeling of OB-projecting cells (CTB-555) and somatic viral labeling of GABAergic neurons. Bottom, coronal slice through the APC/AONp (1.7 mm anterior to Bregma). CTB produced a classic labeling of cortico-bulbar glutamatergic cells in L2 and L3 in the APC. Double-labeled cells (arrowheads) were mainly found in the AONp, located at the border between the APC and OT. Starred cells are magnified in the top panels. This observation was seen in n=3/3 animals. Blue, DAPI.

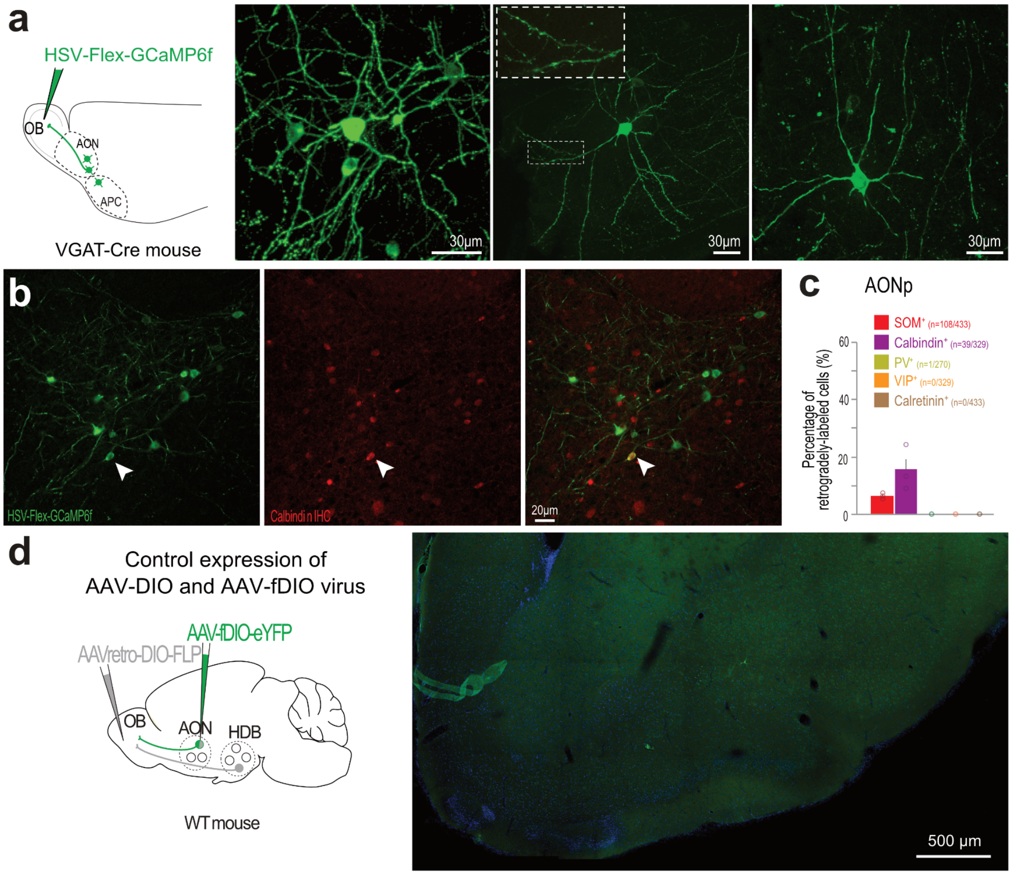

Supplementary Fig. 3. Further characterization of the OB-projecting GABAergic cells. **a**, Representative examples of retrogradely-labeled cells quantified in Figure 2c (n=5 mice). Middle and right panels show spiny (see inset, detail of the boxed region) and unspiny neurons from the AON/APC. **b,** Examples of IHC for calbindin labeled OB-projecting GABAergic neurons (VGAT-Cre x HSV-Flex-GCaMP6f in the OB) in the AONp (arrowhead) and quantified in (c). **c,** Percentage of the OB-projecting GABAergic neurons in the AONp that expressed classical markers for GABAergic neurons (n = 3 mice). Data is mean ± sem across mice. Circles are individual mice. n numbers are the number of double-labeled cells counted for each molecular marker amongst the total analyzed retrogradely-labeled cells. **d,** Representative example image showing specificity control for our INTRSECT labeling. Same as figure 2h, but in wild-type mice. A single neuron was labeled in the entire AOC (right; this observation was confirmed in n=2/2 animals). Source data are provided as a Source Data file.

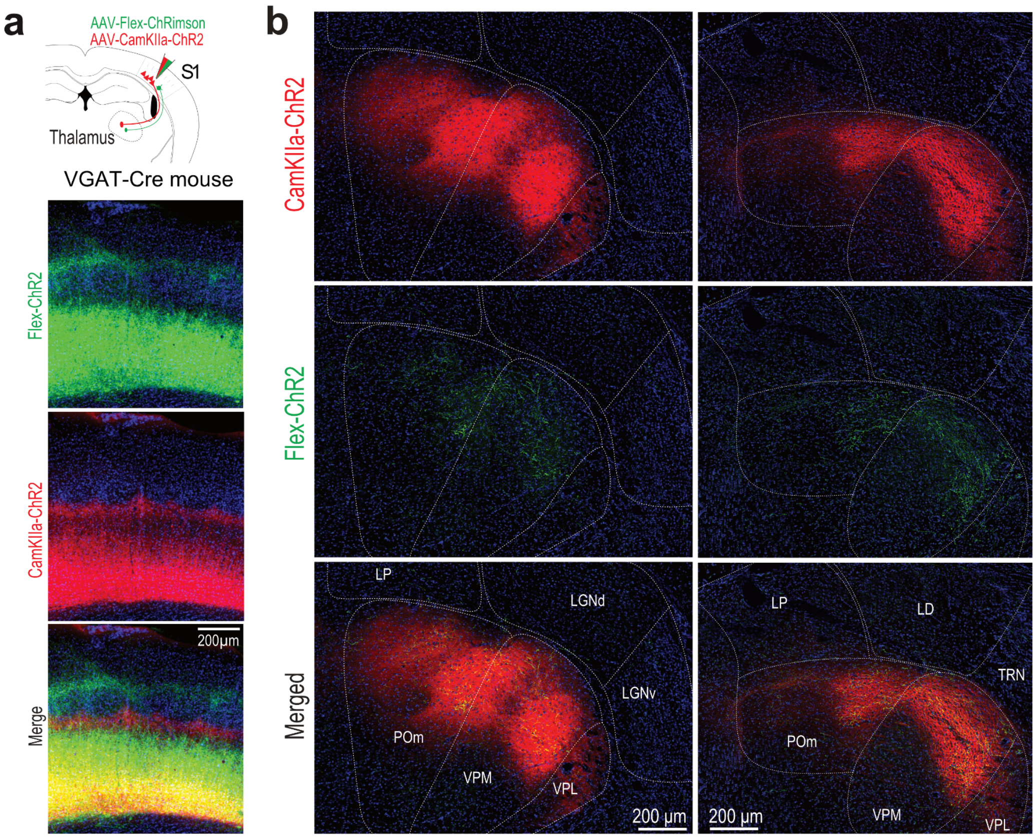

Supplementary Fig. 4. The primary somatosensory cortex (S1) also sends GABAergic projections back to the somatosensory thalamus nuclei. a, Representative example pictures of anterograde labeling of S1 barrel field glutamatergic (ChR2-mCherry) and GABAergic (ChR2-eYFP) axons in the sensory thalamic nuclei. Right, Injection site in L5/6 of S1, barrel field. Blue, DAPI. **b**, Representative examples of Glutamatergic (red) and GABAergic (green) axons across 2 sections (from n=2 mice) through the sensory thalamic nuclei. Blue, DAPI. Thalamic nuclei: LD: latero-dorsal LP: lateral posterior; POm: posteriomedial; VPL: ventral posterolateral; VPM: medial ventral posteriomedial; TRN: thalamic reticular nucleus.

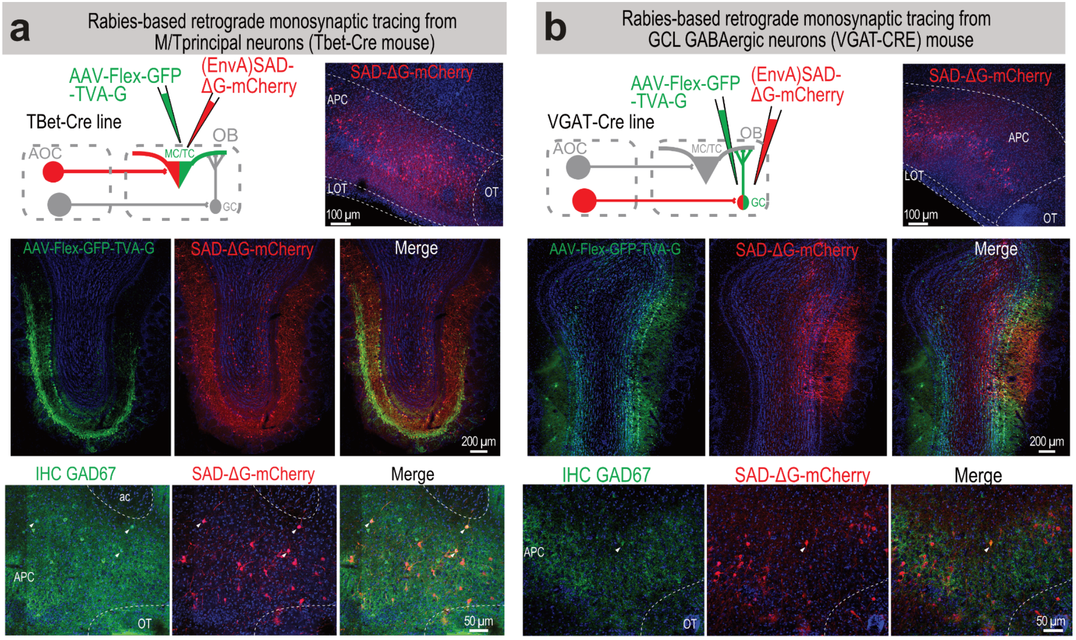

Supplementary Fig. 5. Monosynaptic retrogradely-labeled neurons from genetically identified OB neuron populations. a, Rabies-based transsynaptic retrograde tracing from MCs and TCs ("starter cells"). An AAV encoding the Cre-dependent GFP, avian virus receptor (avian tumor virus receptor A, TVA) and rabies glycoprotein (G) was injected into the OB of Tbet-Cre mice that express Cre recombinase specifically in MCs and TCs. Subsequent injection of G-deleted envelope protein from avian ASLV type A (EnvA)-pseudotyped rabies virus ((EnvA)SAD-ΔG-mCherry) into the OB resulted in transsynaptically retrogradely labeled cells in the olfactory cortex (top, right). Bottom, representative example of IHC for GAD67 revealing that some AOC neurons projecting onto MCs and TCs are GABAergic (arrowheads; this observation was reproduced in n=2/2 animals). **b,** Same as **a** but with GCL interneurons as starter cells (injections in the GCL of VGAT-Cre mice). Bottom, representative example of some double-positive cells sparsely found in the AOC (bottom, arrowhead; this observation was reproduced in n=2/2 animals).

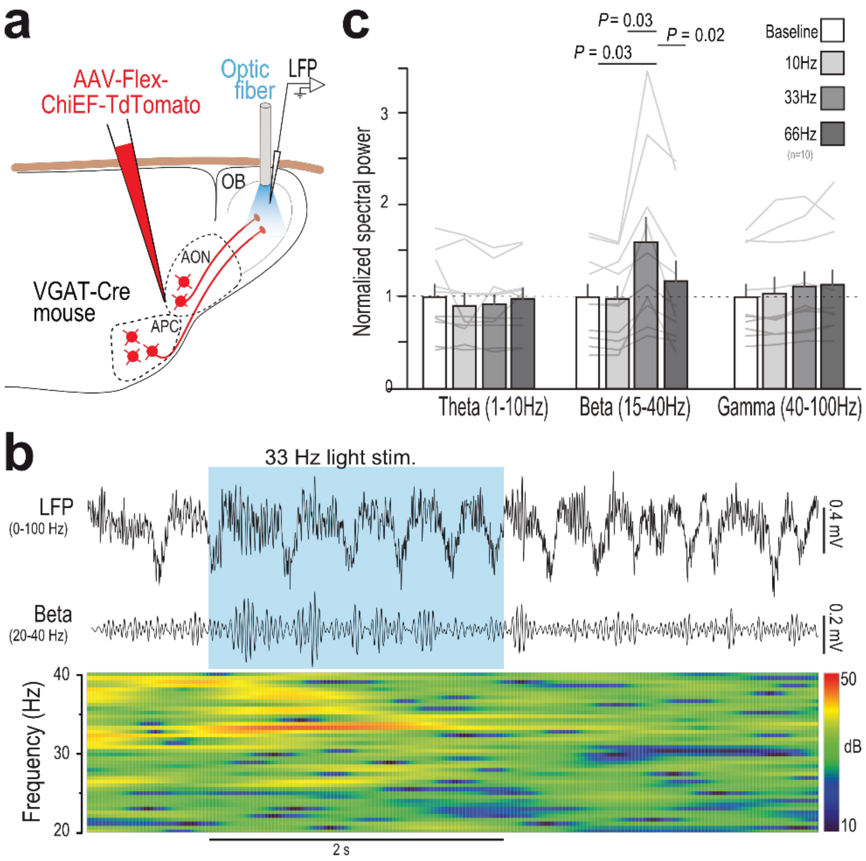

Supplementary Fig 6. Cortico-bulbar GABAergic axon stimulation increases beta oscillations. a, Awake OB LFP recordings while GABAergic cortical axons were optogenetically stimulated through an optic fiber positioned in the dorsal OB in awake mice. **b**, Example broadband (1-100 Hz, up) and beta-filtered (20-40 Hz, middle) LFP trace recorded in the OB. Blue box: 33 Hz light stimulation (2 s). Bottom, corresponding time-frequency spectrogram of the LFP signal in the beta band. **c**, Quantification of the LFP band power (theta, beta and gamma) during 10 Hz, 33 Hz or 66 Hz stimulation patterns (n = 10 recording sites from 6 mice). Interaction between LFP band and stimulation patterns was significant (repeated measures two-way ANOVA, $F(6,54)=8.08$, $P=10^{-6}$). Within the beta band, 33 Hz stimulation only had a significant effect (RM one-way ANOVA, $F(3,37)=9.875$, $p=0.0054$; Tukey's post-hoc test). Data presented as mean ± sem. Gray lines, repeated measures from individual recording site. Source data are provided as a Source Data file.

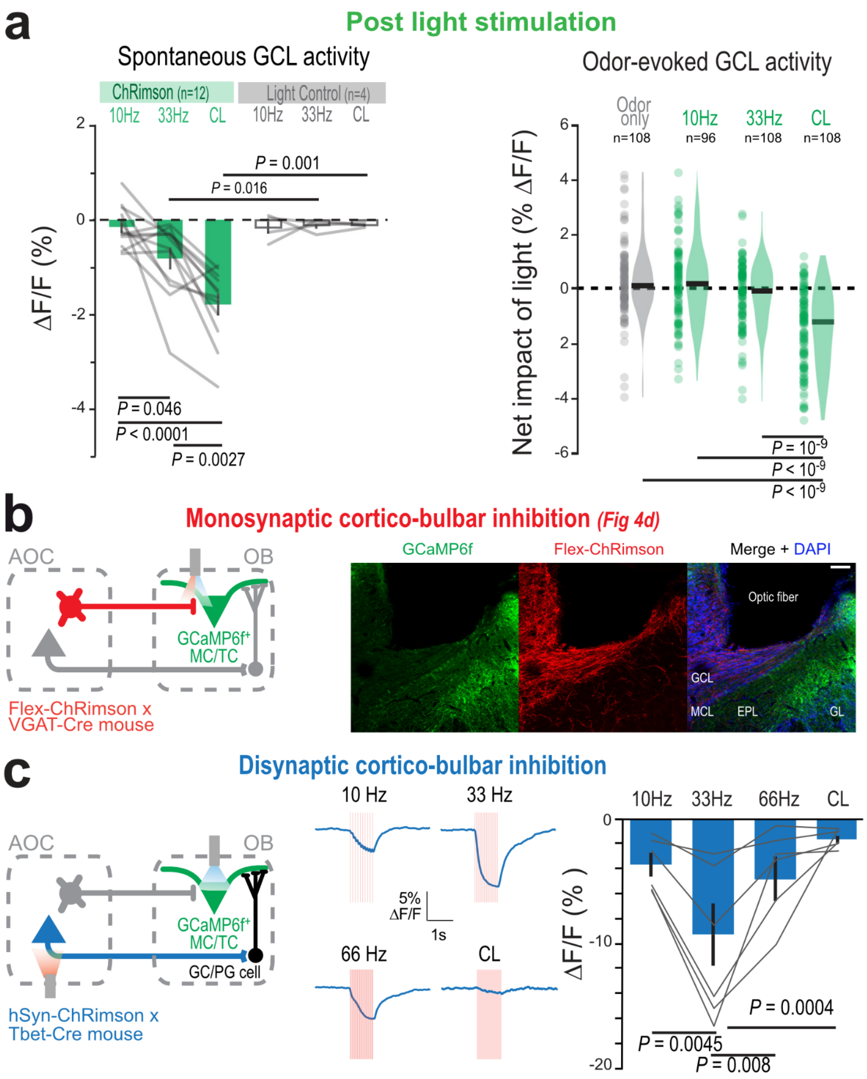

Supplementary Fig. 7. Further characterization of cortical GABAergic inhibition in the OB neuron populations. Inhibition induced by light- stimulation of ChRimson+ GABAergic AON/APC axons in the OB persisted 1 s after light stimulation offset. Left, spontaneous activity (RM-One-way-ANOVA with Tukey's post-hoc test, F(2,22)=20.64, P<0.0001). Right, odor-evoked activity (One-way-ANOVA with Tukey's post-hoc test). Data presented as mean ± sem; gray lines, individual mice. Violin plots are ks density estimates; black bar is median; circle, individual odor-recording site pair. **b**, Stimulation of GABAergic cortical feedback produces monosynaptic inhibition onto MC/TC populations (Fig. 4d). Right, Representative confocal image showing GCaMP6f expression in MCs and TCs, ChRimson-tdTomato in GABAergic cortical

axons and the placement of the optic fiber above the lateral MCL and EPL, further analyzed in Figure4d. Blue, DAPI. Scale bar, 200 µm. **c**, Left, Non-specific cortical feedback stimulation produces disynaptic inhibition onto MC/TC populations. Non-specific cortical feedback consists mainly in glutamatergic feedback. MC/TC population responses were collected utilizing fiber photometry in freely moving mice. In these experiments, we used Tbet-Cre mice to restrict GCaMP6f expression to MCs/TCs. Middle, example averaged responses of MC/TC populations across different cortical feedback stimulation patterns. Right, U-shaped inhibition magnitude with increasing light stimulation frequency, up to CL. Cortical feedback stimulation at 33 Hz produced significantly more inhibition than any other stimulation pattern (RM-One-way ANOVA with Tukey's post-hoc test, $F(3,15)=10.89$, $P=0.0005$, $n = 6$ recordings sites in 3 mice). This contrasted with the monotonic increase of inhibition magnitude with increasing stimulation frequency of cortical GABAergic feedback, up to CL (see Figure 4d). Data presented as mean ± sem; Gray, repeated measurements from same recording site. Source data are provided as a Source Data file.

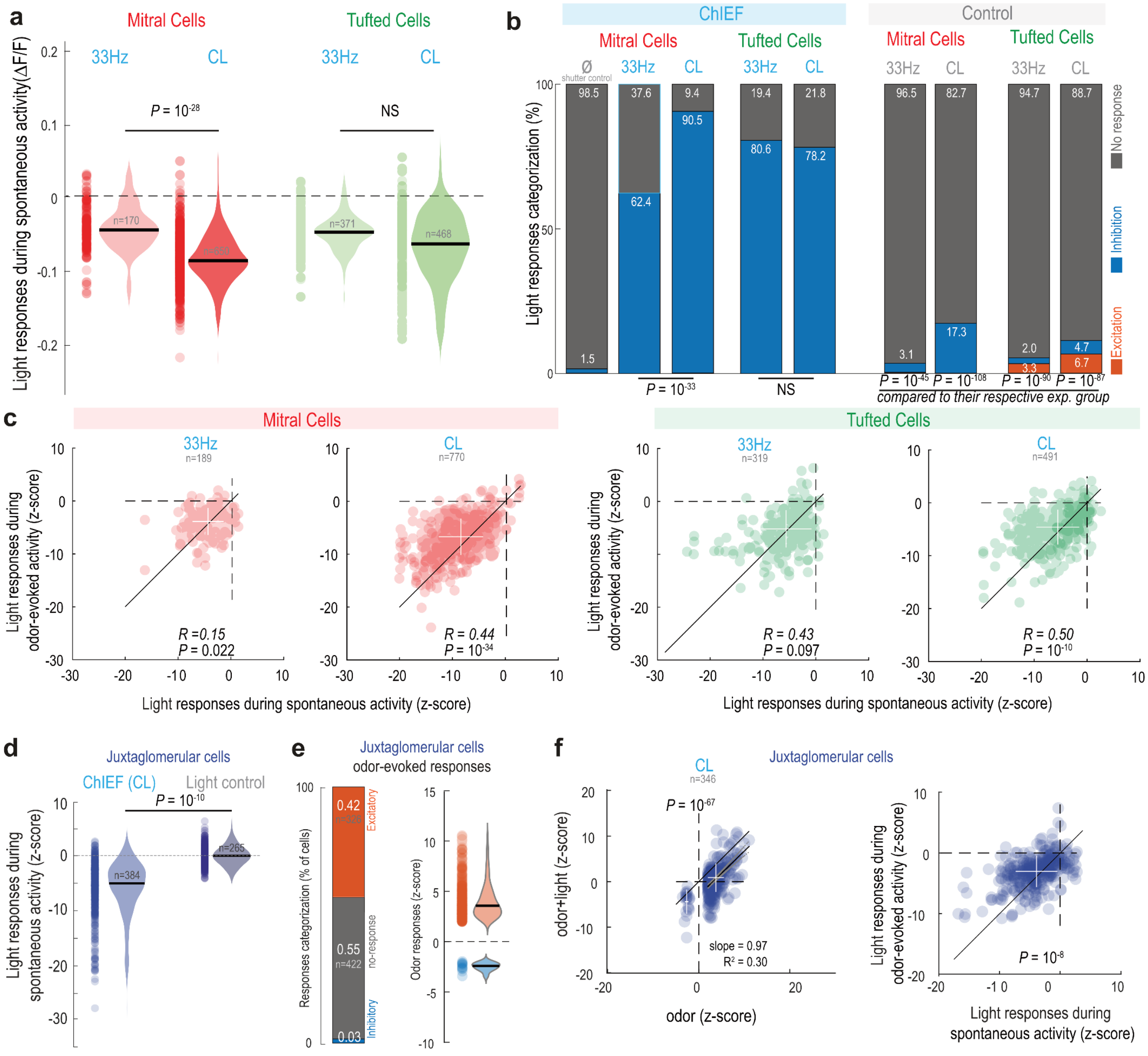

Supplementary Fig. 8. Additional analysis of cortico-bulbar inhibition of OB output neurons. a, ΔF/F0 light responses to 33 Hz and CL for MCs (left) and TCs (right) during spontaneous activity. All experimental groups were significantly higher than their respective light control (two-sided t-test, $P << 0.001$ for all comparisons). CL induced greater inhibition than 33 Hz stimulation in MCs, but not TCs (two-sided t-test). Violin represents ks-density estimation of the data. Black bar, median; circle, individual cell. n as in Fig. 5d. **b**, Categorization of light responses to optogenetic stimulation (left) and light control (right) for MCs and TCs during spontaneous activity. Light caused significantly more significant inhibition in all experimental

groups compared to their respective light control (χ^2 test). CL produced significant inhibition in a greater proportion of MCs, but not TCs (χ^2 test). Note that control CL illumination on MCs induced signification inhibition in a sizable proportion of MCs. n as in Fig. 5d. **c**, Inhibition of spontaneous and odor-evoked activities were weakly correlated (Spearman's correlation coefficient). In odor-responsive MCs (left) and TCs (right), light inhibition of spontaneous activity was slightly stronger than during odor-evoked (two-sided paired t-test), suggesting that the effect of cortical GABAergic feedback stimulation is context-dependent. White cross denotes mean ± s.d. n as in Fig. 5f. **d**, CL illumination induced a significant inhibition of JG spontaneous activity (z-scored) in the presence of ChIEF compared to the "light control" (One-way ANOVA, $F(10,3865) = 456.7$, $p = 0$, with Tukey's post-hoc test). **e**, Categorization (left) and magnitude (right) of the odor-evoked responses in JG cells. Violins are ks-density estimates of the data. Black bar, median; circles, individual cell-odor pair. **f**, Effect of CL stimulation on odor-evoked responses in JG cells. Left: Odor-responses were significantly dampened upon CL stimulation (two-sided paired t-test). Right: Inhibition was slightly greater on spontaneous activity than during odor-evoked (two-sided paired t-test). Source data are provided as a Source Data file.

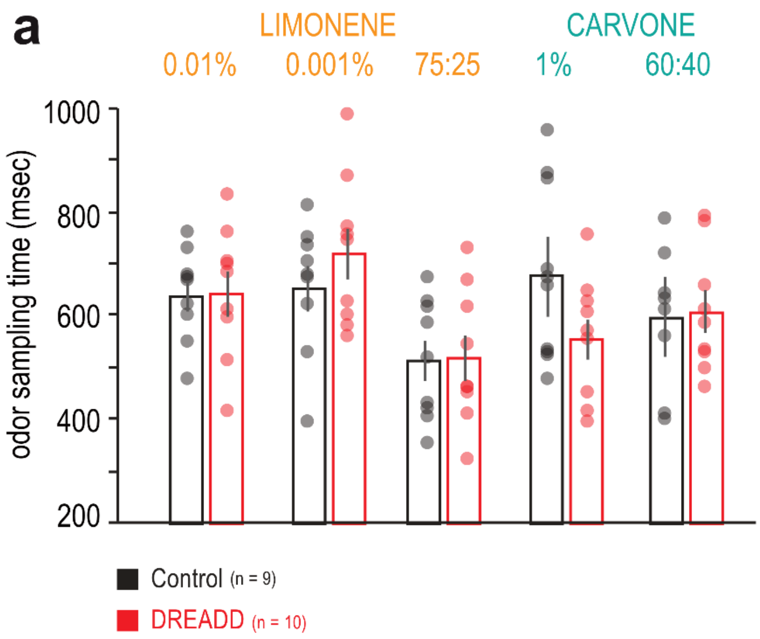

Supplementary Fig. 9. Additional analysis of the behavioral effects of GABAergic cortico-bulbar axon silencing. Mean odor sampling time for correct trials in sessions with mean performance on the last three blocks superior or equal to the criterion level (85%) for control (black) and inhibitory DREADD groups (red). Data presented as mean ± sem. Circle, individual mouse. Source data are provided as a Source Data file.

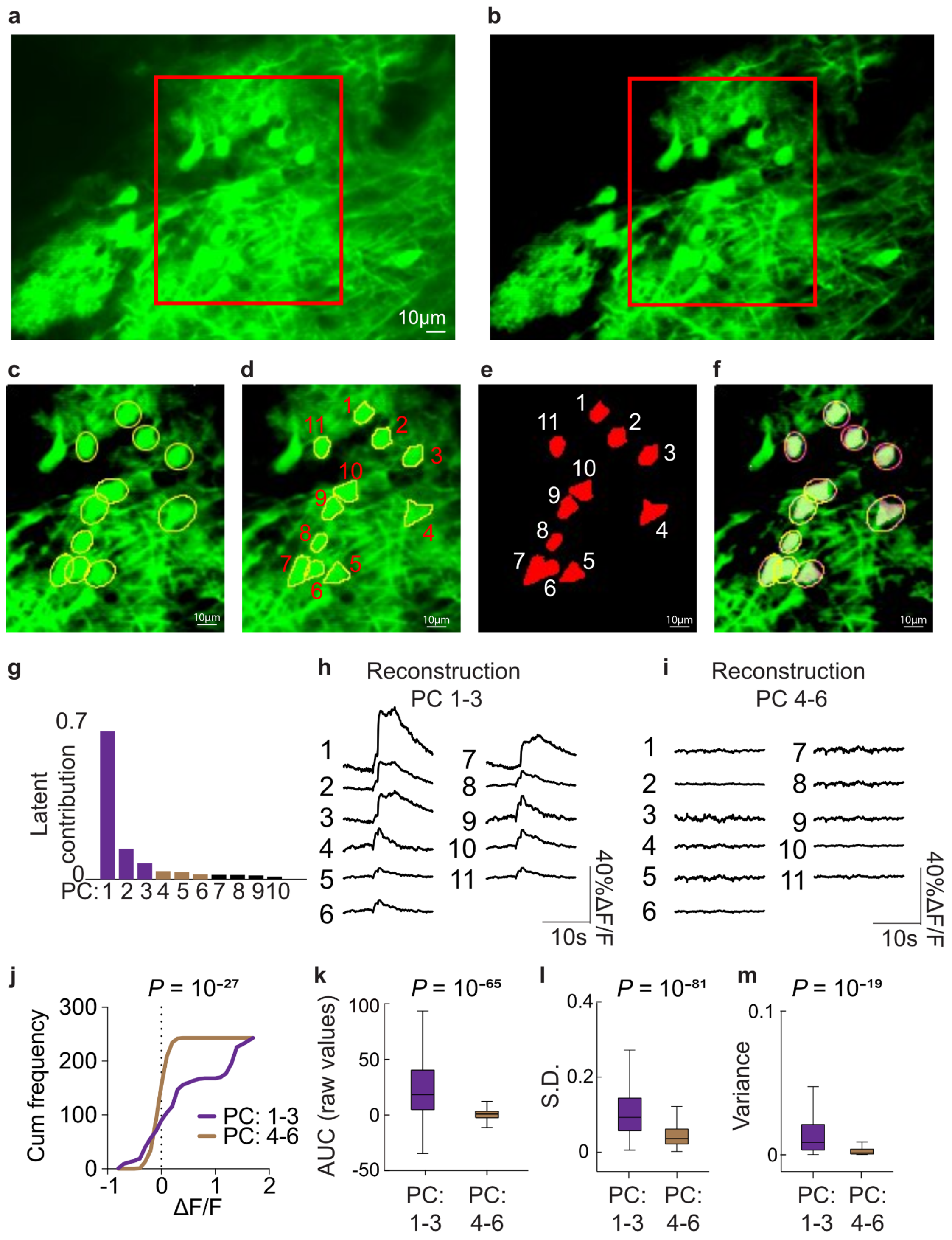

Supplementary Fig. 10. Protocol for neuronal activity data extraction using awake 2-photon imaging. a, Representative example of a field of view (maximum projection) from motion corrected raw images from a 2-photon recording session and further analyzed in the next panels (a-i). This example is part of the data

quantified in (j-m; n = 768 MCs). **b,** Principal component analysis (PCA) reconstruction of the same field of view in **a** to eliminate the background noise and allowing visualization for regions of interest (ROIs) selection. In **a** and **b**, the red boxes indicate the highlighted area in panels **c-f. c**, Manual selection of ROIs after the PCA-assisted reconstruction of the image series. **d,** ROI boundaries were redefined using a second PCA inside each ROI. For illustration purposes only, we show here the ROI reconstruction using the 1st PC only. Note that the new boundary only encompasses pixels smaller than the original elliptical ROIs. **e,** The ROI shape after the reconstruction using PCA inside the elliptical ROIs. **f,** Superimposition of the original manually drawn elliptical ROIs and the PCA-corrected ROIs. **g,** Eigenvalue contribution plot indicating that the first 3 PCs inside the ROI capture most of the variation in the dataset. PC1-3 were used to define the ROI boundaries in our study. Traces from the PCA-corrected ROIs in **d** using either PCs 1-3, capturing the most significant variability in the original dataset (**h**) or PCs 4-6, capturing the 'noisy' contributions in the dataset (**i**). Traces are from the ROIs in **e. j,** Cumulative frequency of the ΔF/F values using PCs 1-3 (purple) or PCs 4-6 (yellow). Note that the ΔF/F values from PCs 4-6 reconstruction are close to 0 and are significantly different from the ΔF/F value distribution from PCs 1-3 reconstruction (Kolmogorov-Smirnov test, n = 247 MCs). Area under the (AUC) as a measure of activity (**k**), standard deviation (**l**) and variance (**m**) of the ΔF/F values for the reconstructed data from PCs 1-3 are significantly higher than those contributing to the noisy pixels, PCs 4-6 (two-sided paired t-test, n = 768 MCs). Box plots are median and 25–75 percentile box. Whiskers extend to the most extreme data not considered outliers. Source data are provided as a Source Data file.

	Rm (MOhm)	Latencies (ms)	Rise time (ms)	Tau (ms)
**GC**	875.90 ± 117.30 (n = 33)	3.04 ± 0.27 (n = 31)	0.40 ± 0.04 (n = 17)	9.64 ± 1.85 (n = 15)
**dSAC**	268.60 ± 55.71 (n = 10)	2.88 ± 0.31 (n = 8)	0.43 ± 0.09 (n = 7)	10.35 ± 1.77 (n = 6)
**MC**	72.96 ± 7.26 (n = 19)	4.26 ± 0.42 (n = 13)	1.01 ± 0.16 (n = 10)	9.20 ± 1.60 (n = 6)
**TC**	73.27 ± 10.31 (n = 11)	4.74 ± 0.18 (n = 11)	0.87 ± 0.11 (n = 12)	10.11 ± 2.36 (n = 9)
**eTC**	149.30 ± 29.18 (n = 16)	3.33 ± 0.40 (n = 9)	0.70 ± 0.22 (n = 8)	5.88 ± 2.71 (n = 5)
**PG cell**	584.20 ± 46.71 (n = 29)	n/a	n/a	n/a
**sSAC**	264.60 ± 50.78 (n = 9)	n/a	n/a	n/a

Supplementary Table 1. Membrane resistance (Rm) and kinetics of the light-evoked IPSCs in the post-synaptic neurons. Data presented as mean ± sem.
