# Supplementary material for: Long-range GABAergic projections contribute to cortical feedback control of sensory processing
Source: Nat Commun. 2022 Nov 12;13:6879. doi: 10.1038/s41467-022-34513-0 (PMC9653434; doi:10.1038/s41467-022-34513-0)
Supplement: Supplementary file 3 — Reporting Summary [file 41467_2022_34513_MOESM3_ESM.pdf]

nature research

Corresponding author(s): Gabriel Lepousez
Camille Mazo
Pierre-Marie Lledo

Last updated by author(s): September 15, 2022

Reporting Summary

Nature Research wishes to improve the reproducibility of the work that we publish. This form provides structure for consistency and transparency in reporting. For further information on Nature Research policies, see our Editorial Policies and the Editorial Policy Checklist.

Statistics

For all statistical analyses, confirm that the following items are present in the figure legend, table legend, main text, or Methods section.

n/a	Confirmed	
☐	☒	The exact sample size (n) for each experimental group/condition, given as a discrete number and unit of measurement
☐	☒	A statement on whether measurements were taken from distinct samples or whether the same sample was measured repeatedly
☐	☒	The statistical test(s) used AND whether they are one- or two-sided<br>*Only common tests should be described solely by name; describe more complex techniques in the Methods section.*
☒	☐	A description of all covariates tested
☒	☐	A description of any assumptions or corrections, such as tests of normality and adjustment for multiple comparisons
☐	☒	A full description of the statistical parameters including central tendency (e.g. means) or other basic estimates (e.g. regression coefficient) AND variation (e.g. standard deviation) or associated estimates of uncertainty (e.g. confidence intervals)
☐	☒	For null hypothesis testing, the test statistic (e.g. F, t, r) with confidence intervals, effect sizes, degrees of freedom and P value noted<br>*Give P values as exact values whenever suitable.*
☒	☐	For Bayesian analysis, information on the choice of priors and Markov chain Monte Carlo settings
☒	☐	For hierarchical and complex designs, identification of the appropriate level for tests and full reporting of outcomes
☐	☒	Estimates of effect sizes (e.g. Cohen's d, Pearson's r), indicating how they were calculated

Our web collection on statistics for biologists contains articles on many of the points above.

Software and code

Policy information about availability of computer code

Data collection	Zeiss Zen 2.6 Blue (Apotome immunofluorescence microscopy), Zeiss Zen Black (Confocal microscopy), Spike2 9.2 (in vivo electrophysiological recordings & fiber photometry imaging), Prairie View Software 5.4.64.100 (2-photon imaging), Elphy (electrophysiological acquisition)
Data analysis	Spike2 9.2 (fiber photometry imaging data analysis), MATLAB 2018a (2-photon imaging data analysis and computational model), ImageJ 1.53t (Immunofluorescence), Imaris 9.1.2 (analysis of immunolabelled punctas), IgorPro 8 with Neuromatic package (analysis of electrophysiological traces), GraphPad Prism 8 (statistical analysis).<br>The code used for imaging data analysis is available at : https://github.com/camille-lab/GABAergicOlfactoryFeedback

For manuscripts utilizing custom algorithms or software that are central to the research but not yet described in published literature, software must be made available to editors and reviewers. We strongly encourage code deposition in a community repository (e.g. GitHub). See the Nature Research guidelines for submitting code & software for further information.

Data

Policy information about availability of data

All manuscripts must include a data availability statement. This statement should provide the following information, where applicable:

- Accession codes, unique identifiers, or web links for publicly available datasets
- A list of figures that have associated raw data
- A description of any restrictions on data availability

Source data are provided as a Source Data file, available with the paper.
Processed imaging data is available online :
https://zenodo.org/record/7050088#.YxXaSHbMJD8 (https://doi.org/10.5281/zenodo.7050088).
The code used for imaging data analysis is available at :

https://github.com/camille-lab/GABAergicOlfactoryFeedback
Additional datasets generated during and/or analysed during the current study are available from the corresponding author on reasonable request.

Field-specific reporting

Please select the one below that is the best fit for your research. If you are not sure, read the appropriate sections before making your selection.

☒ Life sciences ☐ Behavioural & social sciences ☐ Ecological, evolutionary & environmental sciences

For a reference copy of the document with all sections, see nature.com/documents/nr-reporting-summary-flat.pdf

Life sciences study design

All studies must disclose on these points even when the disclosure is negative.

Sample size	In the figures legends are indicated the number of subjects used in each experimental condition. No statistical methods were used to predetermine sample size. We used our previous experience, availability and feasibility required to obtain statistically significant results as well as previous publications in the field to estimate our sample size.
Data exclusions	Animals in which post-hoc histological examination showed that viral injection or implanted optic fiber were not in the correct location were excluded from analysis. For in vivo photometry imaging, recordings with low fluorescence signals or displaying movement/laser artifacts were discarded from the analysis. For behavior, animals which did not perform the 200 trials in requested time period following drug injection were discarded from the analysis. The criteria are fully described in the Methods section.
Replication	Multiple independent observations were collected for each experiment and all attempts to replicate the data were successful. The number of observation for each experiments are indicated in the manuscript.
Randomization	Mice were randomly assigned to one of the different groups studied.
Blinding	For behavioral experiments, experimenters were blinded to the viral construct expressed in each mouse.<br>For in vivo imaging, experimenters were blinded to group allocation and animal identity during data collection. For in vivo imaging data analysis, automatic analysis routines was performed with the same script executed for each recording/imaging session and were not subjected to potential bias.

Reporting for specific materials, systems and methods

We require information from authors about some types of materials, experimental systems and methods used in many studies. Here, indicate whether each material, system or method listed is relevant to your study. If you are not sure if a list item applies to your research, read the appropriate section before selecting a response.

Materials & experimental systems

n/a	Involved in the study	
☐	☒	Antibodies
☒	☐	Eukaryotic cell lines
☒	☐	Palaeontology and archaeology
☐	☒	Animals and other organisms
☒	☐	Human research participants
☒	☐	Clinical data
☒	☐	Dual use research of concern

Methods

n/a	Involved in the study	
☒	☐	ChIP-seq
☒	☐	Flow cytometry
☒	☐	MRI-based neuroimaging

Antibodies

Antibodies used

Primary antibodies :
Calbindin D-28k Mouse (monoclonal) 1:2000 Swant 300 (clone name : CB300)
Calretinin Rabbit (polyclonal) 1:2000 Swant 7697
ChAT Goat (polyclonal) 1:200 Millipore AB144P
GAD67 Mouse (monoclonal) 1:1000 Merck Millipore MAB5406 (clone 1G10.2)
GFP Chicken (polyclonal) 1:4000 Abcam ab13970
Parvalbumin Rabbit (polyclonal) 1:2000 Swant PV27
Somatostatin Goat (polyclonal) 1:500 Santa Cruz D20
Somatostatin Rabbit (polyclonal) 1:4,000 Immunostar 20067
VGAT Rabbit (polyclonal) 1:1,000 Synaptic Systems 131-002
Vasoactive intestinal peptide Rabbit (polyclonal) 1:1000 Immunostar 20077
RFP Rabbit (polyclonal) 1:4000 Rockland Inc. 600-401-379

	Secondary antibodies :<br>Alexa488 Goat anti-Chicken (polyclonal) 1:1000 Molecular Probes A-11039<br>Alexa488 Goat anti-Rabbit (polyclonal) 1:1000 Molecular Probes A-11034<br>Alexa488 Donkey anti-Rabbit (polyclonal) 1:500 Jackson 711-546-152<br>Alexa488 Donkey anti-Goat (polyclonal) 1:500 Jackson 705-546-147<br>Alexa568 Goat anti-Rabbit (polyc:onal) 1:1000 Molecular Probes A-11036<br>Cy5 Goat anti-Mouse (polyclonal) 1:1000 Jackson 115-175-166<br>Cy5 Goat anti-Rabbit (polyclonal) 1:1000 Jackson 111-175-144<br>Biotin-conjugated Donkey anti-Rabbit (polyclonal) 1:1000 Jackson 711-065-152<br>Biotin-conjugated Donkey anti-Goat (polyclonal) 1:200 Santa Cruz SC-2042
Validation	The primary antibodies are extensively used for IHC purposes by the scientific community with numerous species-relevant citations on the manufacturer's websites. All manufacturers specifically state selective reactivity with mouse.<br>Validation statements of previous antibodies and previous publications can be found as follows :<br>calbindin: https://www.swant.com/pdfs/Monoclonal_anti_calbindin_d28k_300.pdf<br>calretinin: https://www.swant.com/pdfs/Rabbit_anti_calretinin_7697.pdf<br>ChAT: https://www.merckmillipore.com/PT/en/product/Anti-Choline-Acetyltransferase-Antibody,MM_NF-AB144P<br>GAD67: https://www.merckmillipore.com/PT/en/product/Anti-GAD67-Antibody-clone-1G10.2,MM_NF-MAB5406<br>GFP: https://www.abcam.com/GFP-antibody-ab13970.html<br>PV: https://www.swant.com/pdfs/PV27_Rabbit_anti_Parvalbumin.pdf<br>SOM: santa cruz: https://www.scbt.com/p/somatostatin-antibody-d-20<br>SOM immunostar: https://www.immunostar.com/product/somatostatin-antibody/<br>VGAT: https://www.sysy.com/product/131002<br>VIP: https://www.immunostar.com/wp-content/uploads/20077-2003001.pdf<br>RFP: https://www.rockland.com/categories/primary-antibodies/rfp-antibody-pre-adsorbed-600-401-379<br>In addition to this public data, we also provided validation of the antibody for GFP in Supplementary Fig 1a (absence of staining without GFP). We also validated the antibody against PV and SOM in Supplementary Figure 1c with the help of transgenic mouse lines.<br>The secondary antibodies have been validated by omitting the primary antibody.

Animals and other organisms

Policy information about studies involving animals; ARRIVE guidelines recommended for reporting animal research

Laboratory animals	Animals used in this study were of the species Mus musculus and the strain C57BL/6JRj (purchased from Janvier laboratories). Adult (8-16 weeks old) male and female VGAT-Cre (heterozygotes, Slc32a1tm(cre)Lowl, MGI ID: 5141270), SOM-Cre (Ssttm2.1(cre)Zjh, MGI ID: 4838416), VIP-Cre (Viptm1(cre)Zjh, MGI ID: 4431361), PV-Cre (Pvalbtm1(cre)Arbr, MGI ID: 3590684) and Tbet-Cre (Tg(Tbx21-cre) 1Dlc , MGI ID: J203355; Haddad et al., 2013) were bred at the Institut Pasteur, Paris. Animal were kept in individually ventilated cages, in a 12:12 hour light:dark cycle at room temperature (20-22°C) and 40-60% humidity.
Wild animals	The study did not involve wild aminals.
Field-collected samples	The study did not involve samples collected from the field.
Ethics oversight	All experimental procedures were performed in compliance with animal protocols approved by the Institut Pasteur ethical committee (CETEA #89).

Note that full information on the approval of the study protocol must also be provided in the manuscript.
